# Saliva Spectral Signature and LINE‐1 Methylation in Oral Cells: Impact of Air Pollution in São Paulo State Residents

**DOI:** 10.1155/sci5/6254270

**Published:** 2026-01-15

**Authors:** Adriana Rocha Nunes, Kusai Baroudi, Mohamed Jaber, Liebert Bernardes Carvalho, Giovana dos Santos Toledo, Thiago Martini Pereira, Luis Felipe C. S. de Carvalho, Gilberto Fisch, Rodrigo A. Foganholi da Silva

**Affiliations:** ^1^ Department of Dentistry, Postgraduate Program in Health Sciences, University of Taubaté, Taubaté, São Paulo, State of São Paulo, Brazil, unitau.br; ^2^ Department of Clinical Sciences, College of Dentistry, Ajman University, Ajman, UAE, ajman.ac.ae; ^3^ Department of Health Sciences, Centre of Medical and Bio-allied Health Sciences Research, Ajman, UAE; ^4^ Department of Biomedical Engineering, Postgraduate Program in Biomedical Engineering UNIFESP, São José dos Campos Campus Institute of Science and Technology (ICT), São José Dos Campos, State of São Paulo, Brazil; ^5^ Department of Pathology, Center for Epigenetic Study and Genic Regulation-CEEpiRG, Program in Environmental and Experimental Pathology, Paulista University, São Paulo, State of São Paulo, Brazil, unesp.br

**Keywords:** air pollution, DNA methylation, epigenetics, gene expression, LINE-1, public health

## Abstract

Air pollution, characterized by the presence of pollutants in the air in large quantities, is one of the main factors degrading the quality of life, especially in industrialized urban centers. This study investigated how air pollution affects LINE‐1 methylation and expression in Taubaté and Lagoinha, cities selected for their contrasting characteristics regarding pollution. DNA and RNA samples were extracted to evaluate LINE‐1 methylation and LINE‐1. The bisulfite PCR technique was used to quantify methylation, whereas RT‐qPCR was employed to measure mRNA expression. Saliva spectral analysis was performed using FT‐IR spectroscopy. The results showed a significant difference in the methylation of the L1TD1 gene: In Taubaté, methylation levels were significantly lower, whereas LINE‐1 mRNA expression was higher compared to Lagoinha. Furthermore, spectral analysis revealed small variations in the intensities of phosphate bands in DNA, suggesting structural alterations. The inverse correlation between pollution levels and the methylation status of the LITD1 gene in oral mucosa cells indicates that the concentration of pollutants may contribute to genomic instability promoted by hypomethylation, potentially leading to the development of chronic diseases. These findings provide evidence that air pollution significantly impacts DNA methylation and LINE‐1 expression and alters the chemical composition of saliva, suggesting that these factors may serve as biomarkers for studies on pollution exposure and disease risk. Therefore, it is essential to implement public policies to reduce air pollution and protect health.

## 1. Introduction

Air pollution is determined as the presence of pollutants in the air in large quantities for long periods and has been characterized as one of the main factors in the degradation of the quality of life of populations, as it is an important risk factor for human health, especially in industrialized urban centers. This issue worsens rapidly, primarily due to the unbalanced development of urban spaces and the significant increase in population mobility, leading to higher road traffic levels [1].

The World Health Organization (WHO) reports on six major air pollutants, which can be classified into two categories [2]: the primary pollutants, emitted directly into the atmosphere by sources, such as carbon monoxide (CO), radioactive waste, sulfur oxides (SOx), lead (Pb), and chlorofluorocarbons (CFCs) (WHO, 2023); the secondary pollutants, which result from chemical reactions that occur in the atmosphere between primary pollutants and natural constituents, such as ozone (O_3_), carbon dioxide (CO_2_), and particulate matter (PM), and can promote serious deleterious effects on human health [3, 4].

According to a resolution published in 1990 by the National Environmental Council (CONAMA), an air pollutant is considered to be “any substance present in the air that, due to its concentration, may render it unfit, harmful or offensive to health, inconvenient to public well‐being, harmful to materials, fauna, and flora or detrimental to the safety, use, and enjoyment of the property and normal activities of the community” [5]. The recent version of CONAMA from 2012 introduced important proposals for environmental management in Brazil, including guidelines for air quality monitoring, surveillance programs, and contingency plans for emergencies related to pollution, aimed at protecting public health [5].

In Brazil, a range of air pollutants are monitored, including PM and gases. ANVISA guidelines, through Resolution No. 9, provide technical guidance on indoor air quality standards [6], whereas CETESB (Environmental Company of the State of São Paulo), following State Decree No. 59113/2013, establishes air quality standards that include gradual targets for reducing air pollution (Decree No. 59113/2013), where the concentrations of particles smaller than 10 and 2.5 microns (PM10 and PM2.5) are monitored, in addition to nitrogen oxides (NOx), SO_2_, O3, benzene, and CO gases, aiming to protect public health and maintain a healthy environment [7].

Among air pollutants, PM stands out as one of the main constituents, and properly assessing its biological impact is crucial not only for human health but also for the environment [8]. We know that air pollution mainly affects those living in large urban areas, where road emissions contribute most to the degradation of air quality [9]. Reports conducted in many locations around the world show a correlation between daily ranges of PM concentrations and daily mortality, as fine PM and ultrafine PM appear to be associated with more severe disease because they can invade the deeper parts of the airways and more easily reach the bloodstream [8, 9].

Based on the magnitude of the impact on public health, it is certain that different types of interventions to minimize their deleterious effects, as well as finding safe methodologies to assess their impact on human health, must be considered as people with asthma, pneumonia, diabetes, and respiratory and cardiovascular diseases are especially susceptible and vulnerable to the effects of PM [10]. In this sense, different research groups have demonstrated that the exposure of individuals to stress and environmental contaminants appears to have a direct impact on epigenetic derepression and mobility of transposable elements (TEs), which can lead to the development of diseases [11].

Class I TEs (LINE‐1) are the most abundant TEs in the mammalian genome, comprising almost 20% of the entire human genome. Under normal physiological conditions, the expression and retrotransposition of LINE‐1 elements are suppressed by complex molecular mechanisms, with hypermethylation being the main mechanism [11, 12]. Studies have shown that hypomethylation of LINE‐1 can lead to its transcriptional activation and increased expression [13]. Thus, accumulated evidence indicates that the loss of its methylation and consequent transcriptional activation are directly related to tumor development and progression processes and its methylation status can be used as a potential molecular biomarker in determining the risk, prognosis, and aggressiveness of various types of cancer [14–16].

Regarding epigenetic alterations, hypomethylation events are the most frequent epigenetic alterations and are correlated with the development of different diseases because they are involved in biological processes, in both health and disease conditions [17]. This fact makes it a focus of great interest, mainly because it has been demonstrated that the methylation status of LINE‐1 can be modulated by atmospheric pollutants such as PM2.5 and nitrogen dioxide (NO_2_) [18], which can induce epigenetic alterations in the oral mucosa, thus contributing to the development of inflammatory and neoplastic diseases [19]. The debate on the influence of air pollution and health has become increasingly relevant, as emerging data indicate that exposure to air pollution modulates DNA methylation and that these changes may, in turn, influence inflammation and the development of diseases [20], with cancer being one of the most discussed diseases correlated with air pollutants and hypomethylation of the LINE‐1 region [21].

Therefore, this study evaluated the possible differences in the methylation status of the LINE‐1 promoter region and its transcriptional activation, as well as characterized the salivary chemical profile in individuals living in cities with higher levels of air pollution compared to individuals living in cities with low levels of air pollution, as salivary composition has been widely studied in differential molecular determination for diagnostic purposes of different diseases such as neurodegenerative diseases [22], inflammatory processes [23], autoimmune diseases [24], and mainly as a liquid biopsy for detecting different types of cancer [25]. However, there have been no reports yet of the use of FT‐IR spectroscopy to compare the salivary composition of people living in places with different atmospheric air qualities, only in the determination of water contaminants [26].

## 2. Materials and Methods

### 2.1. Study Setting and Participants

The study was conducted to investigate the associations between the chemical and molecular composition of saliva and the exposure of participants to different concentrations of atmospheric pollutants. For this, two cities in the Paraíba Valley, in the interior of São Paulo, Brazil, where the atmospheric air quality was different, were selected and the salivary spectral signature and the methylation status of the LINE‐1 repeat regions in oral mucosa cells of residents were determined. These cities offer a useful contrast in terms of atmospheric pollutant levels, as indicated by data from CETESB (2013). This observational study was initiated in May 2023 and selected 60 men, 30 residents of the municipality of Taubaté and another 30 residents of the municipality of Lagoinha, both located in the State of São Paulo, Brazil. The age range chosen was 25–45 years, as it presents a lower probability of diseases, and the individuals are in established metabolic balance, with women being excluded due to hormonal oscillation. The study was approved by the Ethics Committee of the University of Taubaté (CAEE: 65647522.5.0000.5501), and participants were invited to participate in the study, either personally by the researcher himself or through collaborators, such as doctors and nurses from the Lagoinha City Hall health unit, as well as dentists from the UNITAU Dental Clinic. Each recruited participant received a detailed introduction and explanation of this study and signed the informed consent.

### 2.2. Baseline Investigation and Data Storage

Information regarding the participants’ clinical history was obtained through a face‐to‐face interview administered by the lead researcher, based on a questionnaire. The main content of the questionnaire was demographic characteristics, lifestyle behaviors, work addresses, and medical history. To ensure the anonymity of the participants, they were identified in the questionnaire using only their initial letters. The data were tabulated in an Excel spreadsheet, with participant names replaced by sequential numbers to ensure anonymity.

### 2.3. Eligibility Criteria

Males aged between 25 and 45, nonsmokers, healthy, and who had lived and/or worked in the city center for at least 5 years were invited to participate in the study. The study excluded male participants who were under 25 and over 45, who were smokers, who did not work or live in the city center, who had chronic respiratory diseases (CRDs), diabetes, cardiovascular diseases, and autoimmune diseases, and who used medication on an ongoing basis. Participants were verbally informed about the research objectives and could choose freely whether to participate. If the participant agreed to participate, their acceptance was documented by signing the free and informed consent form.

### 2.4. Biological Sample

To collect saliva and oral mucosa cells, participants were instructed to rub their tongues on the oral mucosa for 3 min to stimulate salivation and facilitate cell collection. They then discarded the saliva into a disposable plastic cup, which was given to the researcher. The saliva containing the oral mucosa cells was transferred to properly labeled tubes and stored at 4°C. The collected saliva volume in the laboratory was divided into two tubes, which were centrifuged at 3000 rpm for 10 min at 4°C. The supernatant (saliva) was then collected, and the precipitated cells were resuspended: (1) in 250 μL of DNA extraction buffer (10 mM Tris HCl pH 7.8, 5 mM EDTA, 0.5% SDS) for DNA extraction and (2) in 100 μL of TRIzol for RNA extraction. The samples were stored in a biofreezer at −80°C until processing for molecular biology techniques.

### 2.5. Biochemical Analysis

#### 2.5.1. FT‐IR Spectroscopy

Whole saliva samples were evaluated using the Bruker ATR‐FT‐IR Spectrometer equipped with a diamond crystal for spectral data acquisition. To acquire the spectra, 1 μL of whole saliva was inserted under the ATR crystal. After the sample was placed on the crystal, it was allowed to dehydrate for 1.5 min before analysis. Spectral acquisition was performed with 32 scans to obtain the average of the spectra with a resolution of 4 cm^−1^. After each analysis, the sample was removed from the crystal using absorbent paper moistened with Milli‐Q water and then cleaned with 70% alcohol to completely remove sample residues from the crystal, thus avoiding contamination between samples. Thirty‐two background scans of the equipment were performed for each sample analyzed to remove possible interferences. Each sample was analyzed three times.

#### 2.5.2. Spectral Analysis

First, the data were conditioned. This initial step is crucial for the accurate analysis of the biochemical information present in the sample, as the collected spectroscopic signal may contain, in addition to the differences associated with the sample, fluctuations related to atmospheric water vapor. At this stage, the spectra were smoothed with a Savitzky–Golay low‐pass filter and then converted to their second derivative. This step aims to improve the signal‐to‐noise ratio of the spectrum and eliminate baseline distortions caused by sample heterogeneity. Residual differences in the optical path between the samples were corrected by normalizing the spectra. After this process, a spectral restriction was made in the region between 900 and 1800 cm^−1^ for subsequent biochemical analysis of the data. This region was chosen because it encompasses several vibrational modes of biomolecules present in saliva, such as DNA, RNA, proteins, and carbohydrates. After the data preprocessing stage was completed, the actual analysis of the spectroscopic data was performed. At this stage, we sought to assess the biochemical differences between the Lagoinha and Taubaté groups. For this purpose, principal component analysis (PCA) was used to obtain the eigenvalues and eigenvectors. PCA has proven to be a powerful tool for observing small spectral differences within a set of spectra, allowing the identification of which spectral regions present variations in the quantity of biomolecules, as well as changes in the vibrational modes resulting from structural changes in the molecules. Additionally, the area under the bands in the amide I/II, RNA, and DNA regions was calculated. All processing of the spectroscopic data for this work was performed using Python 3, through routines already developed by Prof. Thiago Pereira, a collaborator on the project.

### 2.6. Molecular Analysis

#### 2.6.1. DNA Genomic Extraction and Endonuclease Treatment (gDNA)

The samples previously homogenized in extraction buffer were digested with proteinase K (20 mg/mL) for 16 h at 56°C, and the DNA was then isolated using the phenol/chloroform/isoamyl alcohol method [27]. The quantity and purity of the extracted gDNA were estimated using a spectrophotometry device, using the OD 260/280 (≥ 1.8) and OD 260/230 (≥ 1.0) ratios (NanoDrop 2000, Thermo Scientific, Uniscience). For endonuclease treatment, each sample was divided into three tubes containing the same concentration (400 ng) and incubated at 37°C for 2 h with 1X NE buffer, 40 mM UDP glucose, and 1 unit of the enzyme T4‐β‐glycosyltransferase (T4‐BGT) in a reaction with a final volume of 20 μL. This enzyme specifically transfers the glucose portion of uridine diphosphoglucose (UDP‐Glc) to 5‐hydroxymethylcytosine (5‐hmeC) by the glycosylation reaction, producing the product beta‐glucosyl‐5‐hydroxymethylcytosine, which specifically blocks the activity of *Msp*I endonuclease and causes it to recognize only methylated (5‐meC) and unmethylated CCGG sequences, keeping the CCGG hydroxymethylcytosine (5‐hmeC) regions intact. After inactivation of the T4‐BGT enzyme by incubation at 65°C for 15 min, the samples were digested with the endonucleases *Msp*I (New England Biolabs, Beverly, MA, USA), which recognizes all CCGG sequences (methylated and unmethylated), except CCGG sequences with the beta‐glucosyl‐5‐hydroxymethylcytosine product, and by *Hpa*II (New England Biolabs, Beverly, MA, USA), which recognizes all CCGG sequences with the difference that methylated CCGG sequences (5‐meC) promote the inhibition of its catalytic activity. The same amount of H_2_O (undigested gDNA—100% control) was added to the third tube. All reactions were performed separately with a final volume of 25 μL at 37°C for 2 h.

#### 2.6.2. LINE‐1 Methylation Measurement

The methylation (5‐meC) and hydroxymethylation (5‐hmeC) patterns of the promoter region of the LINE‐1 type transposase domain containing 1 (***L1TD1***) gene (ID: 54596) were determined in qPCRs containing SYBR Green I Master 2x (12.5 μL), 0.4 μM of specific primers (**F** [5′‐TTC CTT TTT CGC CAG GTA AG‐3′]; **R** [5′‐CGG GCC CCA CGCA CGC A‐3′]), 1.5 μL (25 ng) of treated gDNA (in the three conditions: H_2_O, *Msp*I, and *Hpa*II), and q.s.p of nuclease‐free H_2_O (ultrapure water). The primer sequences were designed in regulatory regions with CpG islands within DNase I hypersensitive regions, regulated by histone modification marks, and with transcription factor binding sites using the Primer3 Input program (version 0.4.0) [28]. The determination of secondary structures and annealing temperatures was analyzed by the Beacon Designer program (https://www.premierbiosoft.com/). All sequences were aligned to the human genome to confirm the chromosomal location by the in silico PCR tool (https://genome.ucsc.edu/). The percentage values for methylation and hydroxymethylation were calculated according to Nestor et al. [29].

#### 2.6.3. RNA Extraction and cDNA Synthesis

Total RNA extraction from oral mucosa cells was performed using the TRIzol/chloroform/isopropanol method. After homogenization of the material in 0.5 mL of TRIzol reagent, the aqueous phase was separated by adding 0.2 mL of chloroform (Merck, Whitehouse Station, NJ, USA) and centrifuging (14.000 rpm) for 15 min at 4°C. At the end of centrifugation, the aqueous phase (supernatant) was collected in properly identified tubes, and the precipitated phase was discarded. Then, RNA precipitation was performed by adding 0.5 mL of ice‐cold absolute isopropanol (Merck, Whitehouse Station, NJ, USA). The samples were incubated at room temperature for 10 min and centrifuged again (14.000 rpm for 15 min). At the end of centrifugation, the isopropanol was discarded by inversion and the precipitated RNA (pellet) was washed with 75% ethanol, resuspended in 50 μL of ultrapure water, and stored at −80°C. The quantity and purity of the extracted RNA were estimated in a spectrophotometric device, using the OD 260/280 (≥ 1.8) and OD 260/230 (≥ 1.0) ratios (NanoDrop 2000, Thermo Scientific, Uniscience). For cDNA syntheses, 1000 ng of previously extracted total RNA was stored in a biofreezer (−80) in the presence of 2 U of the reverse transcriptase enzyme (Superscript II Kit, Invitrogen) (Carlsbad, CA, USA) according to the manufacturer’s recommended instructions. The first‐strand cDNA was generated in a reaction with a final volume of 20 μL containing 500 μM dNTP, 25 μg/mL random primer, 1X first‐strand buffer solution, 40 U of RNAse inhibitor, and 10 μM DTT by the SuperScript II enzyme. Initially, the mixture of RNA, water, and primer was incubated at 85°C for 5 min to promote the opening of possible secondary structures and then the synthesis reaction took place for 120 min at 37°C, with enzymatic inactivation at 85°C for 5 min. After the end of the reaction, the synthesized cDNA was diluted with ultrapure water to a final concentration of 100 ng/μL and stored at −20°C.

#### 2.6.4. LINE‐1 Gene Expression Quantification

To study the expression of the ***L1TD1*** gene (ID: 54596), reactions were performed in duplicate with specific primers (**F** [5′ AAA CAG GGA TGG TAG GGA AAA 3′]; **R** [5′ GGA CAT TGC GAT TTC CAT CT 3′]) and had the ***18S*** gene as endogenous controls (**F** [5′ CGG ACA GGA TTG ACA GAT TGA C 3′]; **R** [5′ TGC CAG AGT CTC GTT CGT TAT CG 3′]). All reactions will have a final volume of 10 μL, containing 5 μL of SYBR Green I Master, 0.4 μM of specific primers, and 1 μL of the synthesized cDNA and ultrapure water. The relative amount of transcripts was determined by the comparative critical threshold method 2^−ΔCt^. In this method, the mean Ct of the target gene is subtracted from the mean Ct of the controls used, resulting in a Δ^Ct^. To calculate the expression, we substituted the Δ^Ct^ value obtained in formula 2^−ΔCt^. The final values were presented as the ratio between the expression of the target gene and the expression of the gene used as a normalizer. The results were expressed as the mean ± standard deviation of 30 independent samples normalized with the control (assigned value 1).

### 2.7. Statistical Analysis

The data obtained regarding the percentage of 5‐meC, 5‐hmeC, and the 5‐meC/5‐hmeC ratio obtained from participants residing in the cities of Taubaté and Lagoinha were analyzed by Student’s *t*‐test using the GraphPad Prism 7 program (GraphPad Software Inc., San Diego, CA, USA) with a significance level of 5%. All qPCR analyses (expression and methylation) were performed in technical duplicate.

## 3. Results

### 3.1. Characterization of the Localities and Participants

Among the cities of the Paraíba Valley, we highlight that the cities of Taubaté and Lagoinha were previously selected due to their representativeness in terms of different environmental contexts and demographic characteristics, as well as for presenting favorable logistics for data and sample collection. As a strategy to characterize the air quality of the preselected cities, we initially chose to search for publicly available quantitative data on the levels of atmospheric pollutants. However, our search revealed that only the city of Taubaté was monitored by the Brazilian regulatory agency (CETESB), thus making it impossible to compare the two cities. In the next step, using the geographic coordinates of the cities of Lagoinha (latitude: −23.0903, longitude: −45.1903, 23° 5′ 25″ south, 45° 11′ 25″ west) and Taubaté (latitude: −23.0309, longitude: −45.5483, 23° 1′ 51″ south, 45° 32′ 54″ west), we were able to access and extract the annual average values of the parameters CO, NO_2_, O_3_, PM_2.5_, and SO_2_ recorded by satellites and deposited in Copernicus Atmosphere Monitoring Service (CAMS) (https://developers.google.com/earth-engine/datasets/catalog/ECMWF_CAMS_NRT). As shown in Figure 1, although the statistical analyses did not reveal significance between the concentrations of the pollutants CO (Figure 1(a)), NO_2_ (Figure 1(b)), CO_2_ (Figure 1(c)), PM 2.5 (Figure 1(d)), and SO_2_ (Figure 1(e)) between the cities, it was possible to verify by comparing the annual values that the concentrations of the pollutants CO, NO_2_, PM_2_, and SO_2_ in Taubaté showed greater accumulation compared to Lagoinha, with no differences being observed between the concentrations of the pollutant O_2_ (Table 1). Furthermore, it was possible to observe that the annual values in micrograms per cubic meter (μg/m^3^) as the sum of the values of the 20 observed years of the main pollutants were higher for the city of Taubaté (Figures 1(f) and 1(g)). When comparing the average annual levels of pollutants with the limits recommended by the WHO, both cities presented CO levels above the recommended values, with Taubaté presenting 8.11 μg/m^3^ more than Lagoinha. For the other pollutants, even though they are not above the recommended concentrations, we see that Taubaté has approximately 2.15 μg/m^3^ of PM_2.5_ and 1.03 μg/m^3^ of SO_2_ more than Lagoinha (Table 2).

Figure 1Comparison of the average annual levels of CO, NO_2_, O_3_, PM2.5, and SO_2_ between Taubaté and Lagoinha. The values referring to the concentrations of atmospheric pollutants were obtained through the open‐access program Copernicus Atmosphere Monitoring Service (CAMS) (ECMWF‐CAMS‐NRT), using the geographic coordinates of Taubaté (−23.0309, −45.5483) and Lagoinha (−23.0903, −45.1903). Graphical representation of the average levels of (a) carbon monoxide (CO), (b) nitrogen dioxide (NO_2_), (c) ozone (O_3_), (d) fine particulate matter (PM_2_._5_), and (e) sulfur dioxide (SO_2_) between the Brazilian municipalities of Taubaté and Lagoinha located in the State of São Paulo extracted for the evaluated period (20 years). (f) Average annual levels of atmospheric pollutants and (g) accumulated values during the 20 years. Results are presented as mean ± standard deviation (2000–2019), *p* > 0.05.

**Figure:**
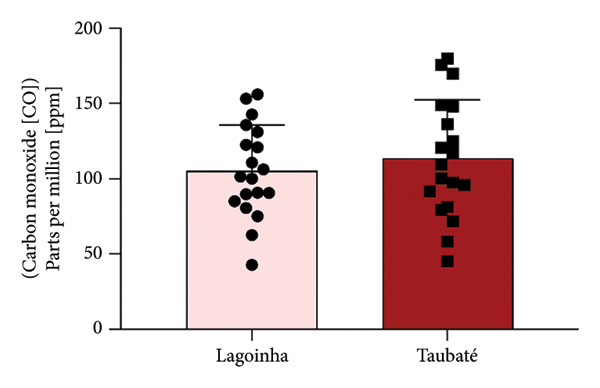
(a)

**Figure:**
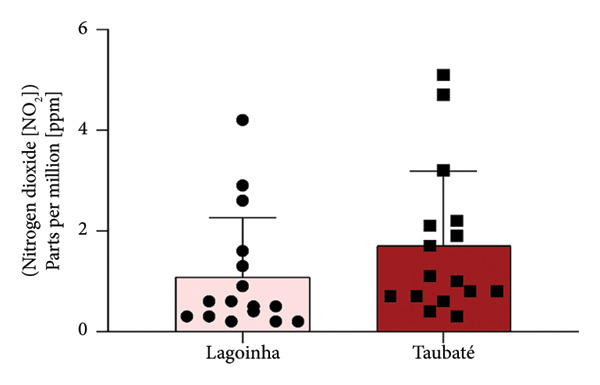
(b)

**Figure:**
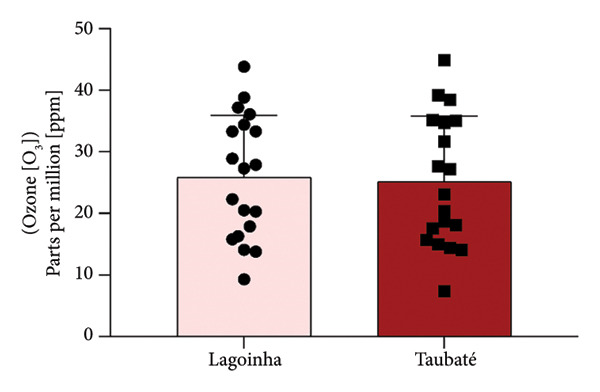
(c)

**Figure:**
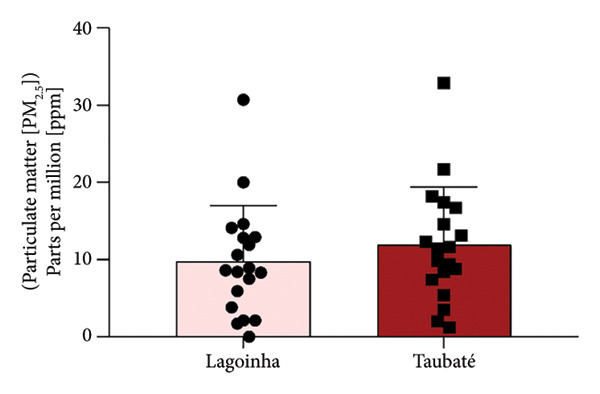
(d)

**Figure:**
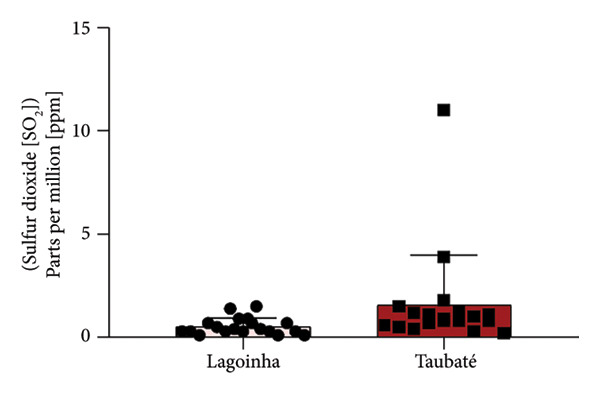
(e)

**Figure:**
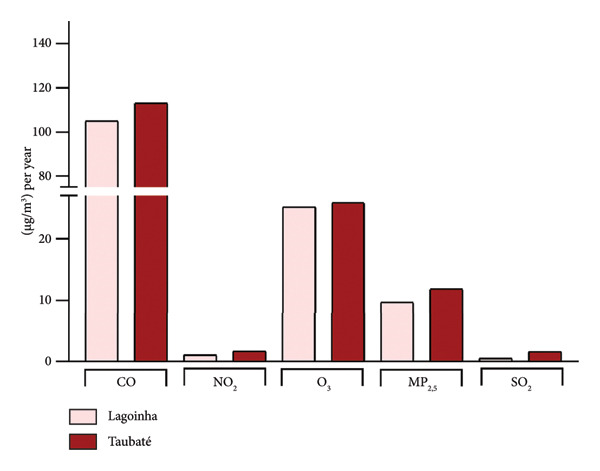
(f)

**Figure:**
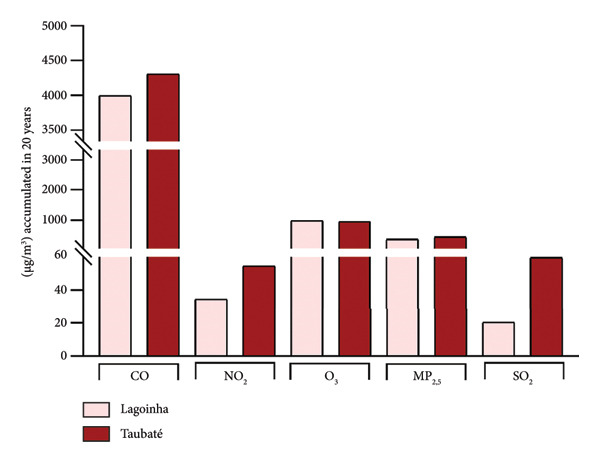
(g)

**Table 1:** Absolute values of atmospheric pollutants.

Years	CO (ppm)		NO_2_ (ppm)		O_3_ (ppm)		MP_2.5_ (ppm)		SO_2_ (ppm)	
	Lagoinha	Taubaté	Lagoinha	Taubaté	Lagoinha	Taubaté	Lagoinha	Taubaté	Lagoinha	Taubaté
2000	110.8	117.2			13.8	14.1	1.7	2.00	0.1	0.3
2001	90.8	100.1			17.9	17.6	20.00	21.7	0.4	0.8
2002	42.8	45.06			15.8	15.7	30.7	32.9	0.1	0.2
2003	153.2	179.7	0.3	0.8	22.3	23.1	8.4	11.4	0.4	1.2
2004	89.8	95.7	0.2	0.7	33.3	31.7	8.6	11.6	0.7	1.5
2005	100.02	58.3	0.2	1.00	27.3	15.34	2.1	8.8	0.3	0.7
2006	131.23	148.2	1.3	2.1	38.8	39.2	12.9	14.6	0.3	0.9
2007	75.02	79.2	0.2	0.4	20.5	20.4	7.5	7.4	0.3	0.8
2008	135.8	175.8	2.9	4.7	14.1	14.4	10.6	18.2	1.5	3.9
2009	85.13	97.6	0.6	1.1	20.3	18.1	3.8	5.4	0.1	0.4
2010	122.6	136.4	0.9	1.7	36.1	35.23	12.8	17.4	0.3	11.23
2011	101.4	109.4	0.6	0.6	34.4	38.4	14.1	13.1	0.9	1.23
2012	80.5	81.4	0.3	0.3	27.9	27.2	8.3	9.4	0.3	0.5
2013	62.5	71.8	1.6	2.2	28.9	27.7	8.9	9.8	0.9	1.1
2014	90.6	91.8	0.5	1.9	43.8	44.9	0.05	1.2	0.7	0.8
2015	106.3	120.5	0.4	0.7	33.3	34.7	2.1	3.5	0.3	0.6
2016	142.7	149.03	2.6	3.2	16.3	18.7	11.9	12.3	0.5	1.1
2017	97.75	125.23	0.5	0.8	37.2	35.2	5.9	8.4	0.7	1.3
2018	156.1	169.8	4.2	5.1	9.3	7.4	14.6	16.7	1.4	1.8
2019	120.9	113.26	1.08	1.7	25.8	27.75	9.74	11.88	0.53	1.57

*Note:* Source: The data were obtained through the Copernicus Atmosphere Monitoring Service (CAMS) (ECMWF‐CAMS‐NRT), using the geographic coordinates of Taubaté (−23.0309, −45.5483) and Lagoinha (−23.0903, −45.1903).

**Table 2:** Comparison of the average annual atmospheric pollutant levels in Taubaté and Lagoinha.

Atmospheric pollutants (μg/m^3^)	Taubaté	Lagoinha	OMS (μg/m³)
NO_2_	1706	1081	40
O^3^	25,857	25,184	100
MP^2,5^	11,884	9731	20
SO_2_	1573	0.536	20

*Note:* Source: State Department of Transit (DETRAN). Population and vehicle fleet in Taubaté and Lagoinha (2023).

Furthermore, it is possible to state that Taubaté, having a significantly larger built‐up area and a population of approximately 317,000 inhabitants, has a higher population density and a smaller green area per capita compared to Lagoinha, which has a population of approximately 4.800 inhabitants and predominantly rural activity and a smaller number of motor vehicles (Table 3). Thus, the lower population density and the absence of large industries in Lagoinha allow us to state that its characteristics contribute to its lower levels of air pollution and better air quality compared to Taubaté.

**Table 3:** Population and vehicle fleet in Taubaté and Lagoinha.

Variable (N.)	Taubaté	Lagoinha
Population	317,915	4953
Cars	180,000	1200
Trucks	15,000	150
Bus	15,000	150

*Note:* Source: State Department of Transit (DETRAN). Population and vehicle fleet in Taubaté and Lagoinha (2023).

### 3.2. Biochemical and Molecular Analyses

#### 3.2.1. Evaluation of the Saliva Spectral Signature

Figures 2(a) and 2(b) show the average absorbance spectrum and its second derivative, after smoothing using the Savitzky–Golay filter. In the average absorbance spectrum (Figure 2(a)), it is possible to observe small differences in the intensities of the bands located in the region between 1000 and 1200 cm^−1^, which are associated with the vibrational modes of the phosphate. These variations may be related to the phosphate groups (PO_2_) present in DNA molecules. Changes are also observed in the amide I/II regions, which correspond to specific vibrations of the chemical bonds in the peptide chains that form the proteins.

Figure 2Characterization of salivary spectral signature by FT‐IR. (a) Second derivative mean spectrum, obtained by a Savitzky–Golay filter, from Lagoinha and Taubaté in the fingerprint region. (b) Mean absorbance spectrum from Lagoinha and Taubaté in the fingerprint region (900–1800 cm^−1^). (c) Mean spectra from each group. The gray bars indicate the wavenumbers with a statistically significant difference using a *t*‐test (*p* < 0.05) in the region between 900 and 1350 cm^−1^. (d) The first three eigenvectors (loading plots) extracted from the principal component analysis of the second derivative spectra.

**Figure:**
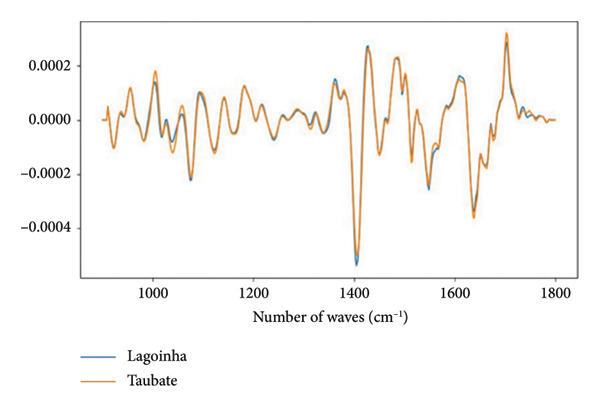
(a)

**Figure:**
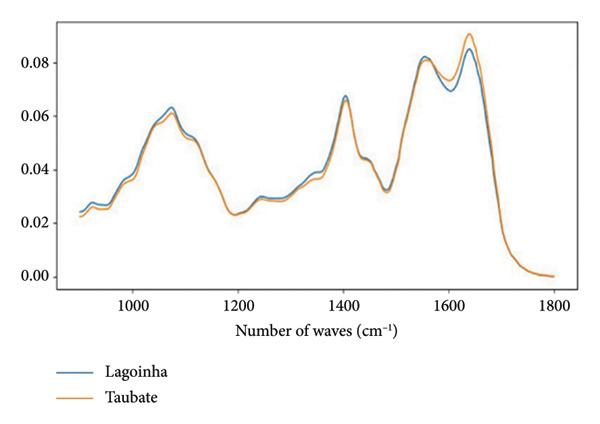
(b)

**Figure:**
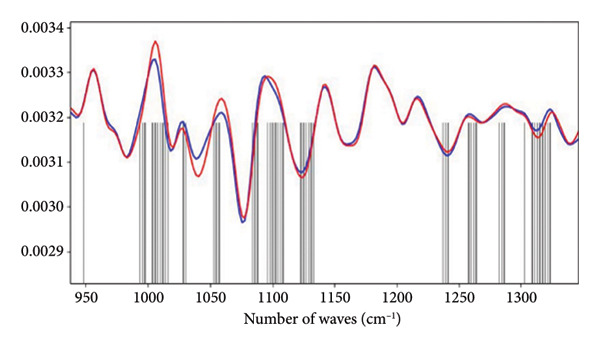
(c)

**Figure:**
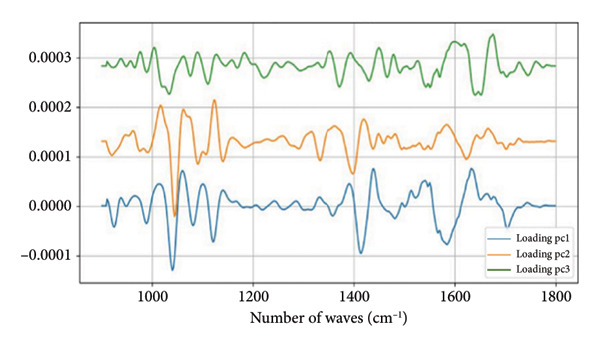
(d)

However, these results cannot be adequately interpreted by observing the absorbance spectrum alone, as the observed bands result from a weighted sum of several very close vibrational modes (sub‐bands). Therefore, it is necessary to analyze the second derivative of these data, followed by the analysis of the loading plots and scatterplots generated from the PCA. Thus, it is possible to note in the spectra converted to the second derivative that in the region between 1000 and 1200 cm^−1^, displacements occur at 1077 and 1240 cm^−1^, which are related to the symmetric and antisymmetric stretching of PO_2_^-^, respectively (Figure 2(b)). The close relationship between the methylation in exactly the found peaks is related to the PO_2_ vibrations of DNA. These findings reinforce the hypothesis of the existence of alterations in the circulating DNA present in the saliva of the residents of Taubaté and Lagoinha. The results discussed above were confirmed by the analysis of the loading plots presented in Figures 2(c) and 2(d).

#### 3.2.2. Characterization of the Transcriptional Profile and Methylation Status of the L1TD1 Gene From Oral Mucosa Cells

As the next step of the study, the methylation status of the *L1TD1* gene and the amount of transcript for LINE‐1 were determined by qPCR. The results presented in Figure 3 show that the buccal cell samples from participants residing in the city of Taubaté presented a higher amount of LINE‐1 transcript compared to those living in Lagoinha (Figure 3(a)). Furthermore, the analyses also revealed that individuals residing in Taubaté have a lower percentage of the 5‐meC methylation mark with no significant changes in the percentage of the 5‐hmeC mark in the promoter region of the *L1TD1* gene (Figures 3(b) and 3(c)). To better understand the epigenetic landscape, the ratio between the 5‐meC/5‐hmeC markers was determined, as this ratio reflects the total methylation status of the promoter region evaluated. In this context, we detected a significant reduction in the 5‐meC/5‐hmeC ratio in samples from residents of the city of Taubaté, confirming the hypomethylation of the promoter region of the *L1TD1* gene (Figure 3(d)).

Figure 3Determination of LINE‐1 gene expression and methylation status of the promoter region of the *L1TD1* gene. (a) The transcriptional profile of LINE‐1 was evaluated by qPCRs in oral mucosa cells. The *18S* gene was used as a normalizer. The DNA methylation status of the *LITD1* gene was investigated by DNA glucosylation by T4‐BGT and *Msp*I and *Hpa*II digestion followed by qPCR analysis with specific primers. The relative methylation levels were presented as (b) a methylation percentage (5‐meC), (c) percentage of hydroxymethylation (5‐hmeC), and (d) 5‐meC/5hmeC ratio determined using the cycle threshold (Ct) method; the methylation results are presented as Ct (*Hpa*II)–Ct (*Msp*I)/Ct (SE), and the hydroxymethylation results are presented as Ct (*Msp*I) Ct (SE). Results were represented as mean ± standard deviation of 30 participants performed in technical duplicate.

**Figure:**
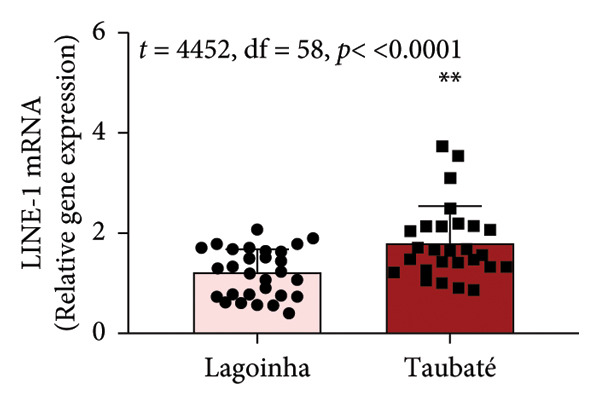
(a)

**Figure:**
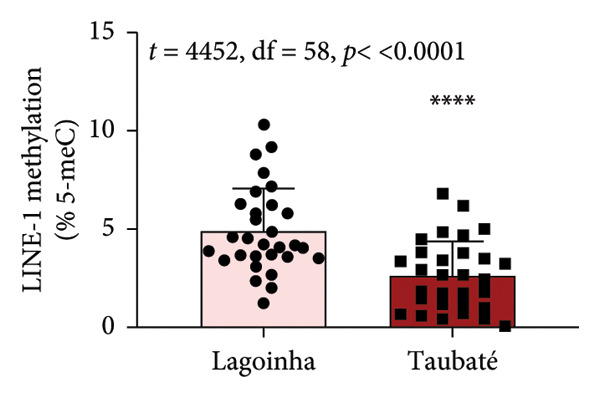
(b)

**Figure:**
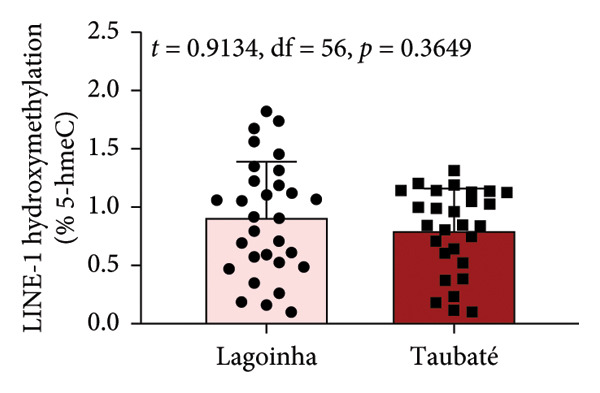
(c)

**Figure:**
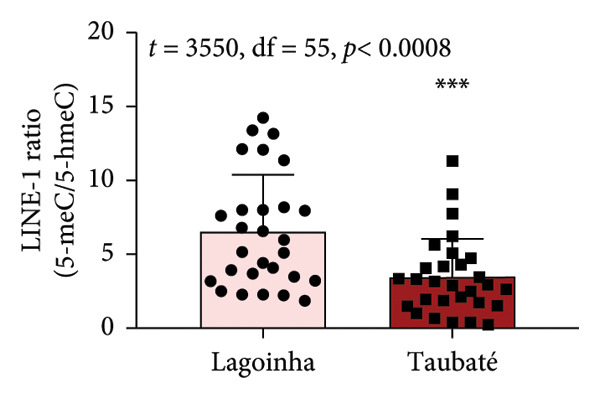
(d)

## 4. Discussion

Atmospheric pollution is a critical factor with profound impacts on public health and the environment, widely documented in studies that link its presence to a range of diseases, including cancer, respiratory, and cardiovascular pathologies [30]. The findings of this study contribute significantly to the understanding of the biochemical and molecular mechanisms underlying exposures to different levels of air quality in distinct regions. Notably, global DNA hypomethylation, particularly in the promoter region of LINE‐1 elements, emerges as an important biomarker of environmental stress and a potential risk indicator for the development of various types of cancer associated with genomic instability [18, 31].

The study was conducted in two main phases: The first involved the selection of locations and participants, whereas the second focused on the execution of laboratory analyses. The choice of the cities of Taubaté and Lagoinha, both located in the Paraíba Valley, was motivated by their environmental and demographic differences, as well as the logistical feasibility for data and sample collection. However, the absence of monitoring stations in Lagoinha presented a challenge, which was overcome by using alternative data sources, such as those provided by CAMS, to ensure the robustness of our conclusions.

This methodological approach allowed us to identify that, although both cities face challenges related to air pollution, Taubaté consistently exhibits higher levels of pollutants such as fine PM (PM_2.5_) and NO_2_. These pollutants are recognized for their harmful effects on respiratory and cardiovascular health. PM_2.5_, for example, is associated with a significant disease burden, contributing to mortality related to cardiovascular and respiratory problems. Similarly, NO_2_, prevalent in urban areas, has a robust correlation with elevated mortality rates, reflecting its negative impacts on the respiratory and cardiovascular systems [32, 33]. The situation in Taubaté, with its dense industrial activity and high vehicle traffic, is exacerbated by its proximity to the Metropolitan Region of São Paulo (RMSP), known for its high pollution levels, which amplify local challenges, especially regarding the formation of O_3_, a secondary pollutant common in urban areas [34].

Conversely, Lagoinha, characterized by a low population density and predominantly rural activities, presented relatively superior air quality, with lower levels of pollutants. However, we observed that O_3_ levels in Lagoinha were slightly higher than in Taubaté, likely due to the lower degradation of this pollutant in rural areas, where the presence of NOx is reduced, thereby decreasing its breakdown [34]. The lack of statistical significance in many pollutant level comparisons reflects the inherent complexity of atmospheric pollution, where interrelated factors, such as seasonal variations and weather conditions, play crucial roles. These findings underscore the need for multidimensional approaches to the formulation of effective public policies [35, 36].

Based on these observations, it was important to explore the potential effects of air quality differences between Taubaté and Lagoinha on the biochemical characteristics of saliva and the epigenetic patterns of oral mucosa cells in participants. Participant selection was carefully made, opting to recruit only men of similar ages. This decision was based on the knowledge that methylation levels in the LINE‐1 region are directly impacted by age, with transcriptional reactivation of these regions being a common phenomenon during aging [37], as well as by the influence of female hormonal fluctuations, which could introduce variability in the results [38]. The relative stability of male hormone levels minimized variability, allowing us to focus on environmental influences and providing a solid foundation for examining molecular data.

The spectral signature of saliva, obtained through FT‐IR spectroscopy, revealed significant differences in regions associated with the symmetric and asymmetric stretching of phosphate (PO_2_^−^), suggesting molecular alterations potentially related to environmental exposure. The average spectra reveal slight variations in absorbance with a peak centered at 1077 cm^−1^, which we attribute to the symmetric stretching of the phosphate (PO_2_) group, likely originating from nucleic acids. Additionally, we observed changes in the peak at 1240 cm^−1^, which we attribute to phosphate stretching, specifically corresponding to the asymmetric mode. Notably, in our data, these two peaks exhibit simultaneous alterations, further reinforcing the hypothesis that these spectral features are primarily due to contributions from nucleic acids.

A key point supporting this interpretation is that, in our PCA, we did not detect any significant differences in the region corresponding to amides I and II (1500–1700 cm^−1^). Given the nature of these vibrational modes, the amide I and II bands are closely linked to peptide bonds. While interactions with phosphate groups (e.g., in phosphorylated proteins) can alter the local environment of these bonds, leading to shifts in the observed frequencies, no such changes were observed in our case. Therefore, based on the evidence presented, the alterations in the phosphate bands without corresponding changes in the amide I and II regions strongly suggest that the observed phosphate‐related modes cannot be attributed to proteins. This further supports the conclusion that these vibrational features are more likely due to nucleic acid contributions rather than protein phosphorylation.

Although salivary composition has been widely studied, this pioneering study explored the comparison between different air quality environments using FT‐IR spectroscopy, demonstrating its value as an instrument in assessing molecular alterations in response to atmospheric pollution. The molecular findings are of considerable importance, indicating that exposure to high levels of atmospheric pollution in Taubaté resulted in a higher expression of LINE‐1 transcripts, mediated by hypomethylation of the *L1TD1* gene promoter region. This hypomethylation, consistently associated with exposure to environmental pollutants, is recognized as an early marker of genomic instability, with implications for the development of various diseases, including cancer [33, 37]. The lower percentage of 5‐meC methylation observed in Taubaté supports the hypothesis that air pollution directly influences epigenetic patterns, promoting hypomethylation in specific DNA regions. The reduction in the 5‐meC/5‐hmeC ratio observed in Taubaté suggests that prolonged exposure to pollutants can significantly alter the epigenetic balance, critically affecting gene expression and genomic stability.

These results highlight the urgency of further investigations into how atmospheric pollution influences DNA epigenetics in oral mucosa cells, contributing to the progression of related diseases. The implications for public health, particularly in urban and industrialized regions, are substantial, indicating that atmospheric pollution may adversely affect not only air quality but also the long‐term health of the population. The connection between LINE‐1 hypomethylation and pollutant exposure reinforces the need for political and environmental interventions aimed at reducing exposure to these pollutants, particularly in densely populated and high‐traffic regions.

## 5. Conclusion

In summary, this study emphasizes the importance of considering atmospheric pollution as a significant risk factor for health, not only for its immediate effects on the respiratory system but also for its long‐term implications on the human genome, mediated by epigenetic mechanisms. Pollution mitigation policies should be prioritized, and further studies are needed to fully elucidate the complex interactions between the environment, the epigenome, and human health.

## Ethics Statement

All experiments were conducted in accordance with the guidelines of the Research Ethics Committee (CAEE: 65647522.5.0000.5501) of the University of Taubaté, Taubaté, SP, Brazil.

## Disclosure

The authors warrant that this research is original, is not under consideration for publication by another journal, and has not been previously published.

## Conflicts of Interest

The authors declare no conflicts of interest.

## Funding

The authors are grateful to Research, Technology, and Innovation Support Foundation of the University of Taubaté (Fapeti) and the National Council for Scientific and Technological Development‐CNPq for Research Productivity Grants—PQ (RAFS—Process: 301498/2022‐9). Luis Felipe C. S. de Carvalho is funded by Fundação de Amparo à Pesquisa do Estado de São Paulo (FAPESP—2017/21827‐1) and by Conselho Nacional de Pesquisa e Desenvolvimento (CNPq/INCT—Interas—406761/2022‐1). Giovana dos Santos Toledo is funded by Fundação de Amparo à Pesquisa do Estado de São Paulo (FAPESP—2025/03886‐7).

## Data Availability

The data that support the findings of this study are available from the corresponding author upon reasonable request.
